# Efficacy of HBV Vaccination in Various Stages of Chronic Kidney Disease: Is Earlier Better?

**DOI:** 10.5812/kowsar.1735143X.751

**Published:** 2011-10-01

**Authors:** Behnam Hashemi, Mitra Mahdavi-Mazdeh, Mohammadreza Abbasi, Seyed Mohammadmehdi Hosseini-Moghaddam, Nadia Hatmi Zinat, Farrokhlagh Ahmadi

**Affiliations:** 1 Public Relations, Tehran University of Medical Sciences, Tehran, IR Iran; 2 Research Center of Iranian Tissue Bank, Tehran University of Medical Sciences, Tehran, IR Iran; 3 Research Center of Nephrology, Tehran University of Medical Sciences, Tehran, IR Iran; 4 Urology and Nephrology Research Center (UNRC), Shahid Beheshti University of Medical Sciences, Tehran, IR Iran; 5 Department of Epidemiology, Tehran University of Medical Sciences, Tehran, IR Iran

## Abstract

**Background:**

Despite improvement in hepatitis B infection prevention through national vaccination programs, implementation of compulsory and thorough blood donor screening, and reduction of transfusion numbers due to erythropoietin administration,hepatitis B remains a major concern in hemodialysis (HD) centers [1]. Compared to aresponse rate of over 90% in the normal population, only 50 to 60% of those with endstage renal disease (ESRD) achieve protective antibody levels following immunization against hepatitis B [2][3]. Various strategies have been developed to overcome the low seroconversion rate in ESRD patients, including co-administering zinc, gamma-interferon,thymopentin, interleukin-2, and levamisole as immunostimulants or adjuvants [3][4],changing the injection mode (intradermal versus intramuscular), or doubling the vaccine dose [5].

**Objectives:**

Previous studies demonstrated that renal failure patients benefit from HBV vaccination; however, not all studies have demonstrated this. Therefore, we compared the rates of seroconversion (hepatitis B surface antibody [HBsAb] titer > 10 IU/mL) in patients at various stages of chronic kidney disease (CKD) (estimated glomerular filtration rate [eGFR] < 60 mL/min/1.73 m2) who received HBV vaccination.

**Patients and Methods:**

A total of 167 patients in 3 different stages of CKD were vaccinated against HBV. Each patient received the vaccine according to a standardized vaccination schedule consisting of 40 μg of the recombinant vaccine “Engerix” at 0, 1, and 6 months.Eight to 12 weeks after the last dose of vaccination, anti-HBsAb levels were measured.

**Results:**

Mean age and eGFR were 57.4 ± 16.5 years and 26.7 ± 14.7 mL/min/1.73 m2, respectively.The overall seroconversion rate was 78%. Although a significant correlation between HBsAb titer and eGFR (r = 0.265, P = 0.001) was observed, in the multivariate analysis using age, CKD stage, diabetes mellitus, and gender as independent variables,the degree of renal function did not significantly contribute to seroconversion. In contrast,higher age (> 60 years) showed a significant negative correlation to seroconversion (odds ratio = 0.22; P = 0.004).

**Conclousions:**

CKD patients of advanced age should be vaccinated against HBV. Although higher eGFR was not associated with improved seroconversion, the persistence of seroconversion was not evaluated; future studies should be conducted to develop recommendations for earlier or later vaccination.

## 1. Background 

Hepatitis B virus (HBV) infection is a widespread but preventable disease. The prevalence of HBV in hemodialysis (HD) patients varies significantly between countries, ranging from very low in developed countries to very high in some developing countries. HD patients are susceptible to infection with HBV and hepatitis C virus (HCV) resulting from blood transfusion, frequent injections, partial immunosuppression, or history of transplantation. Despite improvements in the prevention of HBV infection through national vaccination programs, implementation of compulsory and thorough blood donor screening, and reduction of transfusion numbers due to erythropoietin administration, HBV infection remains a major concern in HD centers [1]. Compared to a response rate of over 90% in the normal population, only 50 to 60% of those with end-stage renal disease (ESRD) achieve protective antibody levels following immunization against HBV [2][3]. Various strategies have been utilized to overcome the low seroconversion rate in ESRD patients, including co-administering zinc, gamma-interferon, thymopentin , interleukin-2, and levamisole as immunostimulants or adjuvants [3][4] as well as changing the injection mode (intradermal versus intramuscular) or doubling the vaccine dose [5].

## 2. Objectives 

Because immune system abnormalities correlate with the degree of renal failure, patients with CKD who do not require dialysis may have a stronger immune system and higher antibody response rate to HBV vaccination than patients who are on renal replacement therapy [6]. Early studies demonstrated that renal failure patients benefit from vaccination; however, not all studies have consistently shown this benefit [7]. Therefore, we designed this study to compare the response rates of patients at different stages of CKD to HBV vaccination.

## 3. Patients and Methods 

Between October 2007 to 2009, 134 cases of CKD (estimated glomerular filtration rate [eGFR] < 60 mL/min/1.73 m2) with evidence of a high creatinine level and chronicity as determined using clinical history or ultrasound findings with negative test results for hepatitis B surface antigen (HBsAg), anti-hepatitis core antibody (anti-HBcAb), anti-hepatitis B surface antibody (anti-HBsAb), and HCV antibody were included in the study. The participants were identified in 2 renal outpatient clinics that offer diagnostic and follow-up services for patients with renal disease. An additional 33 chronic HD patients (prevalent cases) with the described inclusion criteria were added to the group. A total of 167 patients were included in the cohort. Exclusion criteria were current use of immunosuppressive medications and prior history of HBV vaccination or liver disease according to history and liver function tests. Each patient received the vaccine according to the standardized vaccination schedule, i.e. 40 μg of the recombinant vaccine “Engerix” (20 μg in each deltoid region) at 0, 1, and 6 months. Patients received vaccinations through their community health centers or from family physicians, and documents of vaccination were reviewed at each medical visit. In addition to blood tests performed at regular intervals of 0, 2, and 6 months, demographic data were collected from all patients. Laboratory tests included serum creatinine, hemoglobin, electrolytes, albumin, calcium, and phosphate. Medications were also recorded at the start of the immunization schedule and at regular visits. Eight to 12 weeks after receiving the last vaccination dose, the anti-HBsAb level was measured. An anti-HBsAb titer of > 10 IU/L was considered to be a marker of positive seroconversion.Mean creatinine levels during the 3 visits were used to calculate the eGFR using the Modification of Diet in Renal Disease (MDRD) formula. Subjects were categorized into 3 subgroups according to their eGFR: 30–59 mL/min/1.73 m2 (stage 3; 74 subjects), 15–29 mL/min/1.73 m2 (stage 4; 49 subjects), and < 15 mL/min/1.73 m2 (stage 5; 42 subjects). Ten patients with stage 5 disease did not start dialysis therapy before the vaccination schedule was completed.

### 3.1. Statistical Analysis

Descriptive analyses were used to characterize the participant population. Continuous variables were compared by eGFR value using Pearson’s correlation coefficient. Partial correlation was used to explore the relationship between eGFR and HBsAb while controlling for age. Continuous and categorical variables were compared by CKD stage as categorical variables using analysis of variance and chi-square test, respectively. Analyses were conducted using the Statistical Package for the Social Sciences, version 15.0 (SPSS Inc., Chicago, IL). We used the independent-samples t-test and Mann-Whitney U test to compare baseline characteristics between seroconverters and non-seroconverters. A P < 0.05 was considered to indicate statistical significance. A linear regression model was used to identify the factor with the greatest impact on seroconversion.

## 4. Results 

**Figure 1:**
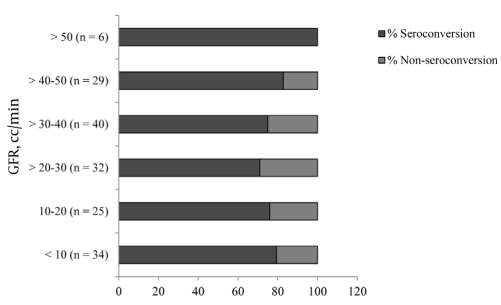
Seroconversion by 10-mL/min Categories of eGFR. P = 0.693 (Pearson Chi-Square)

A total of 167 CKD patients were vaccinated against HBV. Overall, 130 patients were seroconverted following HBV vaccination (seroconversion rate was 78%). Thirty three patients were on dialysis, but the remaining patients with stage 5 CKD did not start dialysis before completing the vaccination protocol. The demographic data of patients according to their CKD stage are listed in Table 1.The mean age was 57.4 ± 16.5 years, the male-to-female ratio was 96/71, and the mean creatinine and eGFR levels were 3.84 ± 3.41 mg/dL and 26.7 ± 14.7 mL/min/1.73 m2, respectively. Differences were observed in hemoglobin, albumin, and weight which were expected due to the impact of the decreased eGFR on these factors. When adjusted for age, there was a correlation between eGFR and HBsAb titer (r = 0.3037, P = 0.001), but the significance of eGFR could not be demonstrated between responders and non-responders (P = 0.547) Table 2. A weak but significant correlation between HBsAb titer and eGFR (r = 0.265, P = 0.001) was observed between sexes (men: r = 0.291, P = 0.005; women: r = 0.04,5 P = 0.713), but this difference was not significant (Zobs = 1.57). In a linear regression model controlled for age, gender, creatinine, weight, cholesterol, and Hb. Level of HBV antibody after HBV vaccination did not reveal a statistically significant difference across different stages of CKD.The HBV antibody titer was also treated as categorical variable. An HBV antibody titer above 10 IU/L was considered as cut-off point for seroconversion. In a multivariate logistic regression model, after adjusting for age, gender, creatinine, weight, and diabetes mellitus (as the cause of CKD), HBV seroconversion was not significantly different between the 3 stages of CKD. In addition, when seroconversion was examined with regard to the eGFR levels using 10-mL/min categories Figure 1, no significant impact could be observed (P = 0.693).

**Table 1:** Patient Demographic Data According to Chronic Kidney Disease Stage

	**GFR a,b, mL/min**					
	**30-59(n =75)**	**15-29(n=50)**	**< 15 mL (n = 42) **			**P value**
			**NoDialysis (n = 9)**	**OnDialysis (n = 33)**	**Total**	
Sex						0.001
Male	53	19			24	
Female	22	31			18	
Age,y,mean ± SD	61.5 ± 14.7	59.7 ± 17.0	54.6±16.4	45.0±13.6		<0.001
Age > 60 y	27/48	21/29	5/9	6/33		0.001
Cause of CKD a						0.107
Diabetes	12	16			19	
Total	75	50			42	
Weight, kg	73.5±12.7	72.1 ± 15.7	60.7±13.1	64 ± 12.0		<0.001
Serum creatinine,mg/dL, mean ± SD	1.7±0.3	2.8 ± 0.61			8.±3.4	<0.001
GFR a, mL/min , mean ± SD	40.5 ± 6.8	22.2 ± 4.6	12.4 ± 1.6	5.9 ± 1.7		<0.001
Hemoglobin, g/dL, mean ± SD	13.3 ± 1.9	11.7 ± 1.7			10.5±1.5	<0.001
HBsAb, mean ± SD	150.6±187.6	115.5±144.3	77.9±81.9	84.4±65.1		0.067
Positive HBs Ab response, No. (%)	60 (80)	38 (76)	6 (67)	26 (79)		0.067

^a^ Abbreviations: CKD, Chronic Kidney Disease; GFR, Glomerular Filtration Rate

^b^ GFR is calculated using the modification of diet in renal disease (MDRD) formula.

**Table 2:** Univariate Comparison and Kidney Function

	**Responders(n=130)**	**Non-responders(n=37)**	**P value**
Sex			0.919
Male	75	21	
Female	55	16	
Age, y, mean ± SD	55.5 ± 16.4	64.5 ± 14.7	0.003
Age > 60 y, No. (%)	61 (46.9)	27 (73)	
Diabetes a, %	22.4	12.9	0.143
Weight, kg	70.4 ± 12.5	70.1 ± 17.9	0.938
eGFR b, mL/min, mean±SD	27.0 ± 15.1	25.36 ± 13.71	0.547
Hemoglobin, g/dL, mean ± SD	12.6 ± 2.0	12.0 ± 1.9	0.158
Cholesterol, mg/dL, mean ± SD	174.4 ± 37.4	196.4 ± 39.8	0.006

^a^ Cause of CKD

^b^ GFR is calculated using the modification of diet in renal disease (MDRD) formula.

## 5. Discussion 

For HBV patients examined in our hospital (age; mean:57.38 years, median: 60.00 years), the MDRD formula is considered to be the most appropriate method for determining eGFR. In a separate study involving more than 30,000 subjects, it was shown that more significant underestimation occurred when using the corrected Cockcroft-Gault equation compared to the MDRD formula in subjects older than 55 years, although a strong correlation was observed between the 2 methods (r = 0.91, P = 0.001) [8]. In our study, although the CKD stage weakly contributed to a higher HBsAb titer, it did not significantly correlate with the seroconversion rate. This finding does not indicate that earlier vaccination is efficacious [9][10]. Although stage 5 CKD patients who were on HD at the time when we started vaccination were included (32/42) in our study, a negative impact of a higher CKD stage on the seroconversion rate was not observed. Our results were similar to those of McNulty et al. who excluded patients on required dialysis [9], or DaRoza et al. who enrolled 14 of 49 patients with stage 5 CKD who required dialysis therapy before completing the vaccination series [6].In contrast to the study by DaRoza et al. no statistically significant difference in seroconversion was observed in different 10-mL/min of GFR categories (P = 0.693) Figure 1. Because the mean eGFR in stage 3 CKD patients in our study was higher than that in DaRoza’s study (41 [30–55] vs. 37 [34–43] mL/min/1.73 m2), the present study is more reliable. Our results agree with those of McNulty, who recommended immunization of patients with progressive kidney disease, preferably in the preterminal stage but not as soon as possible [9]. Bel’eed et al. also found that seroconversion rates were similar in HD patients (66%; 90/136), peritoneal dialysis patients (66%; 36/55), and predialysis patients (68%; 13/19) [10].Serocoversion rate in 30 CKD patients with similar vaccination schedule was studied by Siddiqui et al . They divided the patients according to their creatinine level to mild CKD (2.5 ± 1.1mg/dL, range: 1-3); moderate CKD (4.6 ± 1.5 mg/dL, range: 3.1-6) and ESRD (8.4 mg/dL, range: > 6) . They found seroprotection rate in three groups as 100%,90.1% and 54.5% respectively. [11] However, it should be noted that according to the mean level of creatinine, it seems that moderate CKD in their study may be categorized as stage 4 of CKD and even 5 and it may suffer from relative disparity in groups. As similar creatinine level in different age and weight groups may indicate different level of kidney function.Age is an important contributing factor of responsiveness to HBV vaccination in ESRD patients as well as during the pre-ESRD period [6][9][12][13][14]. Jadoul et al. in a study involving HD patients, showed that seroconversion rates after 12 months were 100%, 75%, and 50% in patients aged < 60 years, 60–75 years, and > 75 years, respectively. Patients with seroconversion were younger (66 ± 14 years) than those without seroconversion (76 ± 9 years) (P = 0.048) [15]. In a meta-analysis of 17 clinical trials of hepatitis B vaccine in patients with ESRD, Fabrizi et al. showed a clear association between advanced age and impaired response to HBV vaccine in ESRD patients (RR: 0.74; P = 0.0139) [13].The success rate of anti-HBV immunization is markedly lower in HD patients (around 60%) than in non-uremics (over 90%). Interestingly, our study showed that patients with stage 3 CKD were older than those with stage 5 CKD, which is in contrast to some reports showing that older patients are typically referred to nephrologists at a later stage [16]. Older patients seek medical advice for different health problems and are referred to nephrologists by other specialists. In addition, physician referrals in Iran are not limited according to laws of insurance agencies, which may be helpful for follow-up of patients. In conclusion, vaccination at an earlier stage of CKD as compared to at a later stage did not result in a significantly higher rate of seroconversion. However, the persistence of seroconversion was not evaluated in this study.
